# Development of the World’s First In Vitro Cancer Epigenome Diagnostic through Biomarker Research for Personalized Cancer Treatment of RAS-wild-type Colorectal Cancer -Secondary Publication - Secondary Publication

**DOI:** 10.31662/jmaj.2025-0074

**Published:** 2025-06-13

**Authors:** Chikashi Ishioka

**Affiliations:** 1 Department of Medical Oncology, Tohoku University Hospital, Sendai, Japan; 2 JR Sendai Hospital, Sendai, Japan

**Keywords:** personalized cancer treatment, biomarkers, in vitro epigenome diagnostic, DNA methylation, genome-wide DNA methylation status

## Abstract

Epigenetic regulation mechanisms such as deoxy ribonucleic acid (DNA) methylation are important for controlling various biological phenomena by regulating gene expression at the genome level. Epigenetic abnormalities are associated with the onset of diseases including cancers. Aberrant DNA methylation is an important epigenetic change in the development and progression of colorectal cancer. DNA methylation in tumor tissues occurs mainly in CpG islands in the promoter regions of genes and inactivates gene functions by negatively suppressing transcription. The CpG island methylator phenotype (CIMP) is an important carcinogenic mechanism in colorectal cancer related to DNA methylation and is involved in approximately 20% of all colorectal cancers. However, CIMP does not always represent the genome-wide DNA methylation status in colorectal cancer. We developed a new method to assess genome-wide DNA methylation status and showed that it is a predictor of the efficacy of anti-estimated glomerular filtration rate (EGFR) antibody drugs and a prognostic factor. This new method has received regulatory approval as a new in vitro diagnostic for predicting sensitivity to anti-EGFR antibody drugs in colorectal cancer.

## Introduction

The epigenome, regulated by deoxy ribonucleic acid (DNA) methylation, histone modification, and micro ribonucleic acid, is important for controlling various biological phenomena such as development, differentiation, growth, and aging across species. Epigenomic abnormalities are associated with the irreversible onset of various diseases, including developmental and aging disorders, neurological disorders, and malignancies. DNA methylation aberrations are an important epigenetic alteration in the development and progression of colorectal cancer (CRC). DNA methylation in tumor tissue occurs primarily at CpG islands of gene promoters and inactivates gene function by negatively repressing transcription. High levels of DNA methylation in CpG islands are referred to as the CpG island methylator phenotype (CIMP) and are thought to be an important carcinogenic mechanism in CRC, occurring in approximately 20%-25% of all CRCs. Our research group has found that genome-wide DNA methylation status is a predictor of sensitivity to anti-estimated glomerular filtration rate (EGFR) antibody drugs, which are standard treatments for unresectable advanced/recurrent CRC (hereafter referred to as advanced/recurrent CRC), and also a predictor of prognosis. Therefore, we have developed a new method to easily determine genome-wide DNA methylation status and obtained regulatory approval for a new in vitro diagnostic that can accurately predict the sensitivity of anti-EGFR antibody drugs for advanced/recurrent CRC.

## Molecular Aberrations and DNA Methylation in CRC

CRC is a prevalent cancer, with a high incidence rate. However, the incidence and mortality rates of CRC vary significantly according to race and ethnicity. Furthermore, the clinicopathologic, histologic characteristics, treatment sensitivity, and prognosis of the cancer differ depending on the location of the primary lesion. Specifically, the location of the primary lesion corresponds to the location of the cecum, ascending colon, and transverse colon on the right side of the body and the sigmoid colon, S-shaped colon, and rectum on the left side. The origins of normal colonic tissue on the left and right sides are different ^(1)^ and many CRCs (over 70%) arise on the left side of the colon. From the perspective of treating advanced recurrent CRC, patients with right-sided CRC have worse overall survival (OS) and recurrence-free survival than patients with left-sided CRC. The left side of the colon is more likely to harbor mutations in the *APC*, *KRAS*, *PIK3CA*, and *TP53* genes, as well as copy number abnormalities and chromosomal instability, with many cases showing deletion or mutation of key tumor suppressor genes ^(2)^. On the other hand, right-sided CRC is slightly more common in women, and there are many cases of *BRAF* mutation, including so-called hypermutation cases where the number of mutations is extremely high despite the diploid state, as well as many cases showing microsatellite instability (MSI-H) due to DNA methylation-induced silencing of the *MLH1* gene promoter region ^(3)^. Furthermore, right-sided CRC is closely associated with the CIMP phenotype ^(4)^.

## Diagnosis of Whole-genome DNA Methylation Status in CRC and Prediction of Sensitivity to Anti-EGFR Antibody Drugs

**Figure 1.**
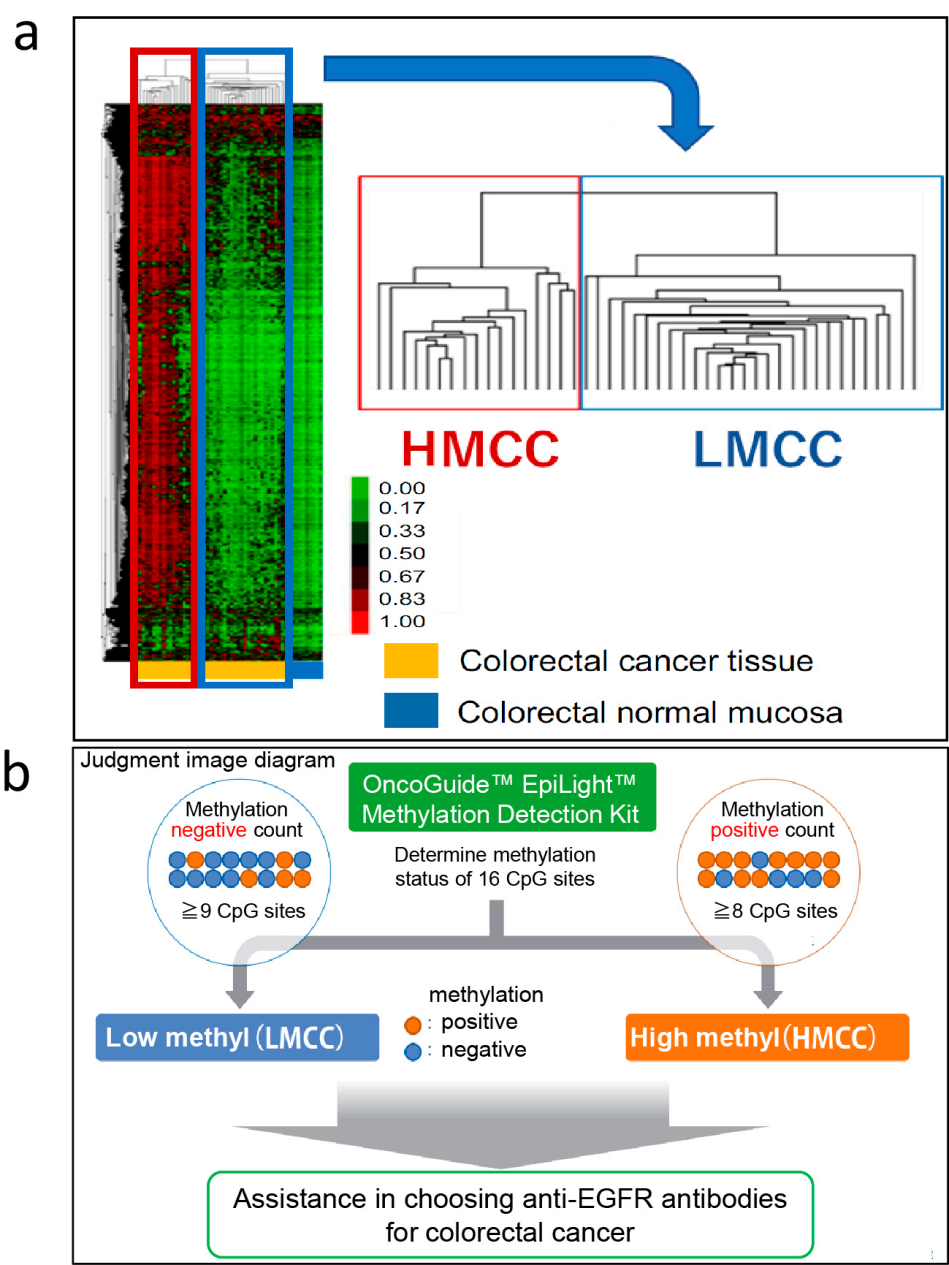
DNA methylation status (GWMS) of the whole-genome in colorectal cancer and EpiLight test. a. Analysis of the methylation status of the entire genome in colorectal cancer using a bead chip and unsupervised cluster analysis, b. Determination of the methylation status using the EpiLight test (taken from the RIKEN GENESIS website). CpG: XXX; EGFR: estimated glomerular filtration rate; HMCC: highly methylated colorectal cancer; LMCC: low methylated colorectal cancer.

Genetic abnormalities in CRC are of paramount importance in guiding treatment strategies for unresectable advanced or recurrent CRC. The presence or absence of *RAS* mutations, *BRAF* mutations, and MSI-H is essential for the selection of combination therapies that include anti-EGFR antibody drugs and BRAF inhibitors as well as immune checkpoint inhibitors. On the other hand, the above-mentioned CIMP has been reported to be useful as a predictor of prognosis and chemotherapy sensitivity in advanced recurrent CRC, but there is no consensus on the usefulness of CIMP as a diagnostic or therapeutic biomarker. Therefore, we focused on the relationship between DNA methylation status and treatment sensitivity to anti-EGFR antibody drugs, which are one of the standard treatments for advanced recurrent CRC. Chemotherapy combined with anti-EGFR antibody drugs is the standard treatment option for advanced recurrent CRC with wild-type *RAS*. However, no reliable biomarkers have been developed to accurately predict the efficacy of anti-EGFR antibody drugs based on epigenomic alterations. We performed genome-wide DNA methylation analysis of tumor tissue DNA from patients with unresectable advanced/recurrent CRC using a bead array (Infinium 450K, Illumina) covering more than 95% of all CpG sites in the genome and found that unresectable advanced/recurrent CRC could be classified into two groups: highly methylated CRC (HMCC) and low methylated CRC (LMCC) (Figure 1a) ^(5)^. This classification was found to be able to extract high methylation CRCs as HMCC at a higher rate than the conventional CIMP. Therefore, we investigated the relationship between genome-wide DNA methylation status (GWMS) determined by bead array analysis and the clinical efficacy of anti-EGFR antibody drugs (response rate, progression-free survival [PFS], and OS). The results showed that GWMS was a predictor of the efficacy of anti-EGFR antibody drugs in the cohort receiving anti-EGFR antibody drugs as third-line treatment (Table 1). The same results were also seen in the cohort of patients receiving anti-EGFR antibody drug combination chemotherapy as first-line treatment (Table 1) ^(6)^.

**Table 1.** Prediction of EGFR Antibody Drug Efficacy Using Whole-Genome Methylation Status and the EpiLight Test.

Detection method for DNA methylation	Treatment line	Patient number	DNA methylation status	Response rate (%)	P value	PFS (M)	P value	OS (M)	P value	Multivariate analyses for PFS and OS
										references
Bead Chip method	3^rd^-line	97	HMCC	3.7	0.01	2.3	<0.001	8.5	<0.001	Independent predictive factor
			LMCC	37.9		6.6		20.9		5
	1^st^ -line	169	HMCC	53.3	0.017	5.7	0.004	31.1	0.019	Independent predictive factor
			LMCC	81.8		13.1		51.4		6
EpiLight method	3^rd^-line	101	HMCC	4.2	<0.001	2.5	<0.001	6.6	<0.001	Independent predictive factor
			LMCC	33.3		5.6		11.8		7
	2^nd^-line	112	HMCC	0	0.02	1.4	<0.001	6.0	0.11^*^	Independent predictive factor
			LMCC	26.6		4.1		11.8		8
	1^st^ -line	137	HMCC	42.9	0.128^*^	4.0	<0.001	13.6	<0.001	Independent predictive factor
			LMCC	71.2		14.3		42.7		9

^*^statistically not significant

## Development of EpiLight and Prediction of Anti-EGFR Antibody Drug Sensitivity and Prognosis

**Figure 2.**
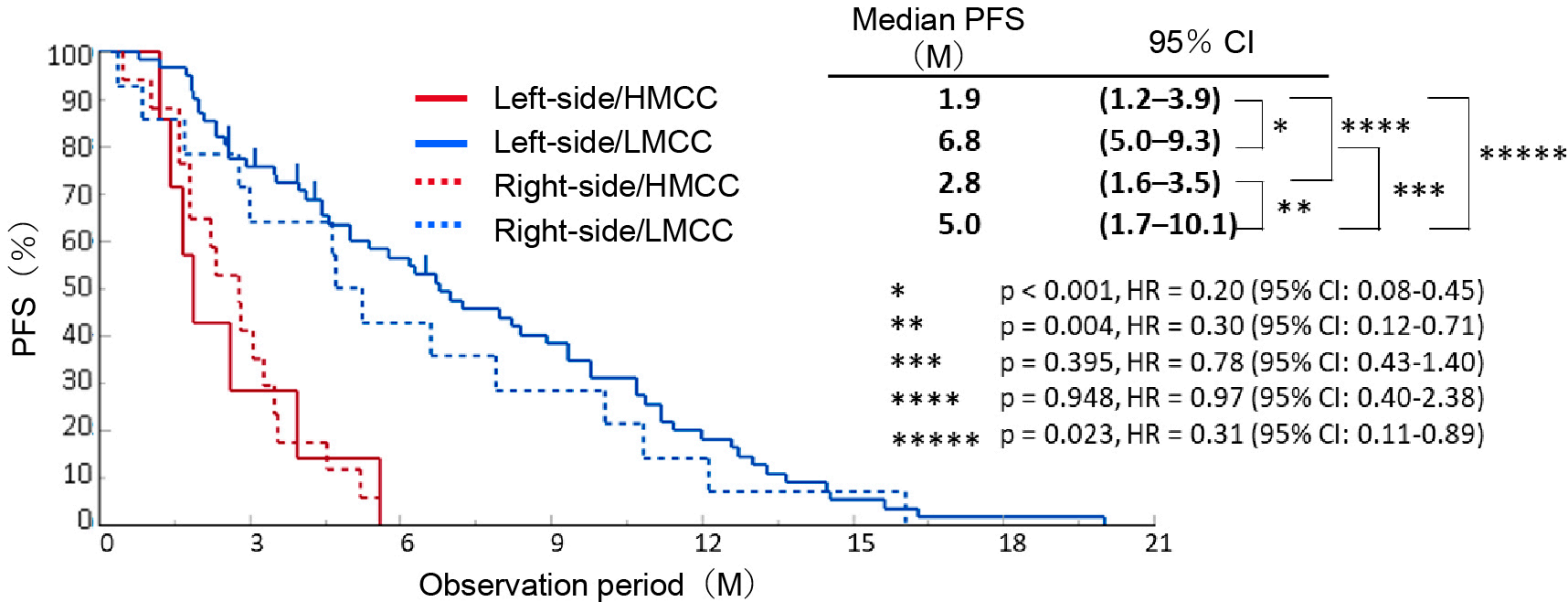
Kaplan-Meier survival curves for PFS by DNA methylation status and primary lesion site. CI: confidence interval; DNA: deoxy ribonucleic acid; HMCC: highly methylated colorectal cancer; HR: hazard ratio; M: month; PFS: progression-free survival.

To apply GWMS-based drug selection in clinical practice, we developed the EpiLight DNA methylation detection kit in collaboration with RIKEN Genesis as an assay for easier classification of HMCC and LMCC ^(7)^. This method uses a real-time polymerase chain reaction to detect the methylation of 16 CpG sites in bisulfite-treated genomic DNA of *RAS* wild-type advanced recurrent CRC patients who received third-line anti-EGFR antibody therapy. It classifies GWMS (HMCC and LMCC) based on the DNA methylation status of the CpG sites in these 16 regions (Figure 1b). PFS after anti-EGFR antibody therapy was compared between four groups of GWMS (HMCC and LMCC) classified by this assay and the primary lesion site (right-sided and left-sided CRC), and the results, showed that GWMS was able to predict the efficacy and prognosis of anti-EGFR antibody therapy with greater accuracy than the location of the primary tumor (Figure 2). In multivariate analysis, DNA methylation status was also an independent predictor of treatment response, independent of other factors including primary tumor location (Table 1). Furthermore, the assay was also able to predict sensitivity to anti-EGFR antibody drugs in cohorts that received anti-EGFR antibody drug treatment in first- and second-line settings (Table 1) ^(8), (9)^. Based on these data, we named the assay the OncoGuide™ EpiLight™ Methylation Detection Kit and applied it to the PMDA for drug approval, which was accepted in May 2023. After one year of review, it was approved on June 21, 2024, as an in vitro diagnostic for use as an aid in selecting treatment for unresectable advanced or recurrent CRC with wild-type RAS gene (as of February 2025 (not yet listed in the National Health Insurance Drug Price List).

### Conclusion

The usefulness of DNA methylation assays as an aid in selecting anti-EGFR antibody drugs was described in the Japanese Society of Clinical Oncology’s “5th Edition of the Guidelines for Genetic Testing in the Diagnosis and Treatment of Colorectal Cancer” in June 2023, one year before the assay was approved for use in Japan ^(10)^. Furthermore, in October 2024, after the drug was approved, a new clinical question was added to the guideline as “Perform DNA methylation analysis as an aid in determining the indication for anti-EGFR antibody drugs.” The recommendation level was “Consideration (expert consensus opinion).” It is expected to be listed in the National Health Insurance Drug Price List shortly, but it is believed that a stronger level of recommendation will be given in the future by providing higher evidence. At present, the primary tumor location is clearly stated in domestic and international clinical practice guidelines as a surrogate biomarker for the selection of anti-EGFR antibody drugs, but in the future, it is expected to be replaced by the evaluation of the methylation status of the entire genome.

## Article Information

This article is based on the study, which received the Medical Award of The Japan Medical Association in 2024. This is a revised English version of the article originally published in Japanese in the Journal of the Japan Medical Association 2024;153(10):1111-4 ^(11)^. The original version is available at https://www.med.or.jp/cme/jjma/newmag/pdf/153101111.pdf. Only members of the Japan Medical Association are able to access it.

### Conflicts of Interest

Riken Genesis, Co., Ltd., Hitachi, Ltd, Chugai Pharmaceutical Co., Taiho Pharmaceutical Co., Daiichi Sankyo Co., Ono Pharmaceutical Co., Janssen Pharmaceutical K.K., Nippon Boehringer Ingelheim Co., Insight Co., Ascent Development Services, PRA Health Sciences, Inc.

### Sources of Funding

The clinical research work was supported by a grant from the Project for Development of Innovative Research on Cancer Therapeutics (P-DIRECT) (grant number 11110018) and the Project for Cancer Research and Therapeutic Evolution (P-CREATE) by the Japan Agency for Medical Research and Development（AMED）(grant number 16770660). The OncoGuide™ EpiLight™ Methylation Detection Kit was co-developed by Tohoku University and RIKEN Genesis, Co., Ltd. by a fund of RIKEN Genesis, Co., Ltd.

### Acknowledgement

We thank our co-investigators, Drs. Kota Ouchi, Makoto Takahashi, Hiroyuki Aburatani, and our collaborative research company, RIKEN Genesis, Co., Ltd., especially Mr. Tatsuro Saito.

### Author Contributions

Chikashi Ishioka substantially contributed to the conception of the work, the acquisition, analysis, and interpretation of data for the work, drafting the work, reviewing it critically for important intellectual content, final approval of the version to be published, and agreement to be accountable for all aspects of the work in ensuring that questions related to the accuracy or integrity of any part of the work are appropriately investigated and resolved.

### Approval by Institutional Review Board (IRB)

Approval code issued by the institutional review board (IRB) and the name of the institution that granted approval: This study “Development of a molecular biomarker for colorectal cancer” (UMIN000005490), which was approved by the Ethics Committee of the Tohoku University School of Medicine (approval number [No.] 2019–1034 and No. 2022–1-737). A part of this study was also approved by the IRB of the Japanese Foundation for Cancer Research (Registry No. 2019–1034).
